# Ambulatory monitoring of activity levels of individuals in the sub-acute stage following stroke: a case series

**DOI:** 10.1186/1743-0003-4-41

**Published:** 2007-10-26

**Authors:** William H Gage, Karl F Zabjek, Kathryn M Sibley, Ada Tang, Dina Brooks, William E McIlroy

**Affiliations:** 1 Toronto Rehabilitation Institute, 550 University Avenue, Toronto, Ontario, M5G 2A2, Canada; 2 Department of Physical Therapy, Graduate Department of Rehabilitation Science, University of Toronto, 500 University Avenue, Toronto, Ontario, M5G 1V7, Canada; 3 Centre for Stroke Recovery, Sunnybrook & Women's College Health Sciences Centre, 2075 Bayview Avenue, Toronto, Ontario, M4N 3M5, Canada; 4 School of Kinesiology and Health Science, York University, 4700 Keele Street, Toronto, Ontario, M3J 1P3, Canada; 5 Department of Kinesiology, University of Waterloo, 200 University Avenue West, Waterloo, Ontario, N2L 3G1, Canada

## Abstract

**Background:**

There is an important need to better understand the activities of individual patients with stroke outside of structured therapy since this activity is likely to have a profound influence on recovery. A case-study approach was used to examine the activity levels and associated physiological load of patients with stroke throughout a day.

**Methods:**

Activities and physiologic measures were recorded during a continuous 8 hour period from 4 individuals in the sub-acute stage following stroke (ranging from 49 to 80 years old; 4 to 8 weeks post-stroke) in an in-patient rehabilitation hospital.

**Results:**

Both heart rate (p = 0.0207) and ventilation rate (p < 0.0001) increased as intensity of activity increased. Results revealed individual differences in physiological response to daily activities, and large ranges in physiological response measures during 'moderately' and 'highly' therapeutic activities.

**Conclusion:**

Activity levels of individuals with stroke during the day were generally low, though task-related changes in physiologic measures were observed. Large variability in the physiological response to even the activities deemed to be greatest intensity suggests that inclusion of such extended measurement of physiologic measures may improve understanding of physiological profile that could guide elements of the physical therapy prescription.

## Introduction

Considerable effort in the rehabilitation process of patients with stroke is orientated towards addressing sensori-motor dysfunction [1,2] and cognitive deficits [2,3]. Although the majority of patients with stroke have concomitant cardiovascular disease, and as such can benefit from aerobic exercise training, the effects of such exercise among these patients is only beginning to be considered in the literature [4,5]. A recent meta-analysis which included seven randomized controlled trials examining the efficacy of aerobic exercise training among patients with stroke reported that there is good evidence to support the use of aerobic exercise among patients with mild and moderate stroke for improving aerobic capacity [6]. Studies that have examined the effects of exercise [7,8] in sufficient dose and intensity have shown that improvements in cardiovascular fitness among individuals with stroke can be comparable to that of healthy, age-matched adults. The benefits of exercise for these individuals include improved cardiovascular and psychological status, and sensorimotor, strength, and endurance measures [9].

The potential importance of activity programs is heightened given the evidence to suggest that individuals with stroke are generally sedentary. Individuals who have had a stroke within the past 14 days and who reside in an acute care hospital spend more than 50% of the day lying in bed, 28% of the day sitting in bed, and 13% of the day engaged in functional activities; therapist contact accounted for only 5.2% of the patient's day [10]. Earlier work reported that activity levels of individuals with stroke residing in a hospital stroke ward were low throughout the day, however the amount of time that had passed since the stroke was not reported [11]. While this work provides some general insight into activity patterns there has been no research to examine daily activity levels and associated cardiorespiratory responses of patients with stroke at the sub-acute stage of recovery in a rehabilitation setting. Traditional rehabilitation programs that focus on improving ability to perform daily function are unlikely to adequately challenge the cardiovascular system of individuals with stroke. Patient heart rates have been shown to reach target ranges considered acceptable for conditioning programs during therapy; however, the length of time in the target range is very brief [5,9]. The brevity of elevated heart rate during therapy, and the low percentage of the day engaged in the therapy program, combined, suggest that the cardiovascular challenge provided to individuals with stroke during a structured rehabilitation program is insufficient to maintain, let alone improve, cardiorespiratory capacity.

Gordon and colleagues [9] suggested, based on previous work by Palmer-McLean and Harbst, and others, that to obtain a cardiovascular training effect, individuals with stroke need to perform cardiovascular exercise at 40% to 70% of heart rate reserve, or 50% to 80% of maximum heart rate, for 20 to 60 minutes per day, 3 to 7 times per week, and that exercise may be performed in multiple 10-minute sessions. Individuals who have had a stroke do appear to benefit significantly from cardiovascular exercise [7-9], and it is clear that they receive very little, if any, cardiovascular benefit from activities during therapy [5]. Clearly more research must be conducted to explore the efficacy and feasibility of cardiovascular exercise after stroke, with particular attention paid to the type and dose of exercise [6]. However, the focus must also be directed to non-therapy related activities since such activities are likely to be an important determinant of the cardiorespiratory fitness profile of individual survivors of stroke. To date, there has been little information to indicate the type and intensity of activities that stroke patients are engaged in during the day when not in therapy. The activities engaged in outside of structured therapy sessions would potentially have a profound influence on the cardiorespiratory status in addition to being an important index of the changes in functional capacity occurring over the course of rehabilitation. The challenge of such work is to be able to assess both activity and the physiologic responses to be able to judge the potential therapeutic benefit of specific daily activities.

The objective of this study was to examine activity profiles and associated cardiorespiratory load of individuals in the sub-acute stage after stroke throughout a day using an ambulatory data collection system. We hypothesized that individual activity levels would not be of sufficient intensity or duration to elicit a cardiorespiratory training effect, even during structured therapy sessions. In addition, we addressed the relationship, within specific cases, between an activity-level classification (rated 0–4) with ambulatory recorded measures of physiological response to activity (heart rate, ventilation rate). We believe that information related to an individual's physiological response to specific activity (whether directly therapeutic or non-therapeutic activity) may be uniquely important for therapists when designing person-specific structured and unstructured activity programs for individuals with stroke.

## Methods

### Participants

Four individuals, all male and ranging in age between 49 and 80 years, volunteered to participate in this study. The participants in this study were selected from a parallel study [12], which examined the feasibility and effects of an aerobic training program among individuals in the sub-acute stage of recovery following stroke. Participants in the current study had recently concluded their involvement in the parallel study. Importantly, these four patients were selected because they represented a range in both age and stroke-related residual deficits in function, allowing a multiple case-study approach to investigating the use of the ambulatory monitoring device, and the individual patient's physiological response to various levels of activity. The inclusion and exclusion criteria for this study were consistent with those of the parallel study. Each participant was screened based on the following criteria: Chedoke-McMaster Stroke Assessment (CMSA) Scale Leg Score [13] between 3 and 6, and the cognitive ability to provide informed consent. The exclusion criteria included: resting blood pressure greater than 160/100 despite medication, other cardiovascular morbidity which would limit exercise tolerance, unstable angina, orthostatic blood pressure decrease of > 20 mmHg, hypertrophic cardiomyopathy, any musculoskeletal impairments which may limit the individual's ability to cycle on a stationary, semi-recumbent ergometer, and ongoing pain which would preclude participation. Person-specific details are reported in Table 1, including information regarding medication use and the location of stroke, NIH Stroke Score, Functional Independence Measure score, peak VO_2 _(VO_2 _was required for parallel study; VO_2 _testing methodology is described elsewhere [12]), and the lower limit of the calculated target heart rate training zone [5]. All participants had experienced a stroke within 2 months prior to testing and were in-patients at the Toronto Rehabilitation Institute at the time of testing. A physician assessed each participant to confirm his medical status prior to entering the study. The local research ethics board approved this study.

**Table 1:** Characteristics of the individual participants.

**Patient ID**		S1	**S2**	S3	**S4**
Age		49	49	78	80
Date of Stroke*		25/04/04	15/07/04	31/01/05	03/01/05
Date of Testing*		28/06/04	23/08/04	23/02/05	15/02/05
Time from stroke to testing (months)		~2	~1	~1	~1.5
Location of stroke		Left interior capsule	Right pontine lacunar	Left cerebellar	Left lacunar
Medication(s)		Atorvastatin Perindopril	Losartan HCTZ Plavix Nifedipine	Diazepam Glycerin Atorvastatin Heparin Sodium ASA	Plavix Rampril HCTZ Cardizem
NIHSS** (adm/disch**)		3/3	5/3	1/1	5/2
CMSA leg score (adm/disch)		6/6	4/5	6/6	3/4
FIM** (adm/disch)		100/115	61/106	74/103	88/107
FIM (locomotion; adm/disch)		6/7	5/5	4/6	2/6
FIM (upper body; adm/disch)		6/7	3/6	5/6	4/6
Resting HR** (day of testing; bpm)		79	71	53	62
Lower limit target HR training zone (bpm)		105	101	77	88
Peak demonstrated HR		113	107	97	110
Peak demonstrated VO_2 _(ml/kg/min)		12.8	10.4	15.2	8.9

**Amount of time in each activity category (AC) [10]**					

**Activity Categories**	**0**	8%	2%	26%	52%
	**1**	51%	21%	28%	13%
	**2**	No samples; see text for explanation			
	**3**	21%	20%	24%	20%
	**4**	20%	57%	22%	15%

* dates are formatted as dd/mm/yyyy

**NIHSS-National Institutes of Health Stroke Score, FIM-Functional Independence Measure, adm/disch-score at admission/score at discharge, HR-heart rate

### Procedures

**Figure 1:**
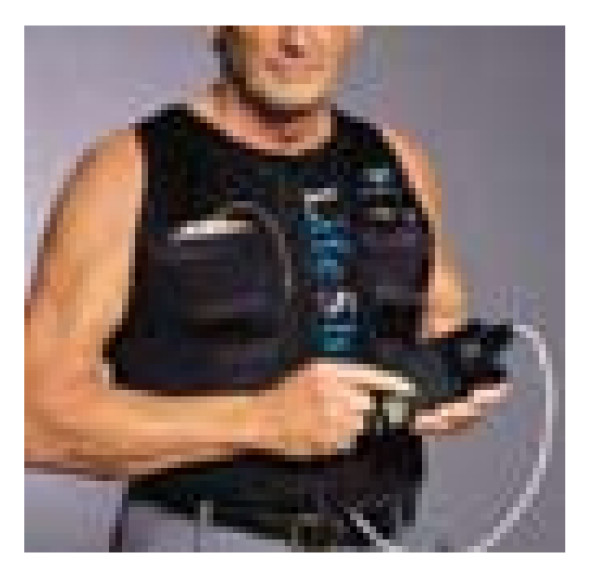
Photograph of the LifeShirt, the data collection system used in this study. ECG and inductive plethysmography bands are embedded in the garment. Data was stored on a PDA (shown).

An instrumented mesh vest (LifeShirt, Vivometrics, Ventura, California, USA) was worn throughout one 8-hour period, from approximately 8 am to 4 pm. The vest was designed to record electrocardiogram (ECG) and plethysmography signals on a dedicated, handheld personal digital assistant (PDA) computer, which was attached to the participant's belt or pants. A picture of the LifeShirt is provided in Figure 1. It should be noted that the LifeShirt is composed of a lightweight mesh material; the weight of the device, including the PDA and battery, is reported on the company's website to be 703 grams. To provide the appropriate context during the collection period, each participant was "shadowed" by two research assistants who were instructed to document any reasonable or notable change in the individual's posture or activity (for example, walking, sitting, climbing stairs), a description of the activity (for example, therapy, reading, watching television, bathroom), and the time at which the activity occurred. At the end of the data collection period, the vest was removed, and the data was transferred from the PDA to a computer for storage and analysis.

### Measures of Interest

**Figure 2:**
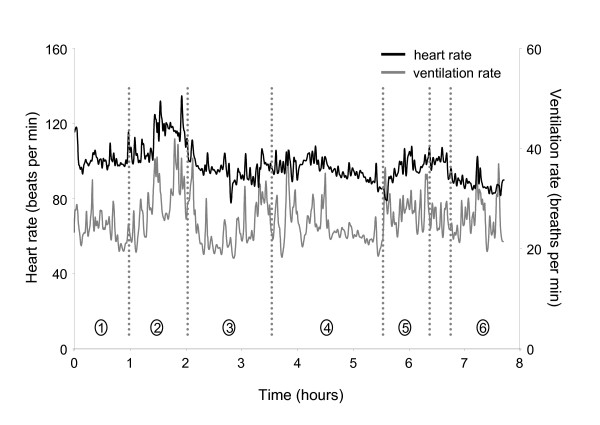
Participant S1; profiles of HR and VR activity throughout the day. Circled numbers refer to the following activities during the associated periods throughout the day: 1, sitting, walking, eating breakfast; 2, physiotherapy (upper extremity weights, floor-level cone placement exercise, treadmill walking, stair climbing); 3, ADLs, walking, prolonged periods of sitting; 4, eating lunch, speech therapy, walking, prolonged periods of sitting; 5, occupational therapy (hand mobility and strengthening exercises); 6, ADLs, sitting while talking with other patients. This patient demonstrated a clear HR response during his physical therapy session (period expanded in Figure 3).

The data recorder sampled the ECG signal at 200 Hz and the plethysmography signal at 50 Hz. Custom software (Matlab, Mathworks, Massachusetts, USA) was used to calculate the heart rate (HR) measure from the ECG signal and to extract ventilation rate (VR) from the plethysmography signal. The HR and VR data were low-pass filtered at 0.5 Hz, for demonstration purposes, and contrasted with the documented activity for one representative participant (Figure 2). All calculations were performed using the raw HR and VR signals.

To reflect periods of sustained HR elevation throughout the day, and the American Heart Association's scientific statement recommendations for exercise [9], we determined the mean HR across a 10 minute window (HR_10_), and serially advanced the window by 1-minute increments to construct a moving-average profile of the HR signal. We determined the individual's resting HR by finding the lowest 1-minute average HR during the collection period; a computer algorithm was used to find the lowest 1-minute average HR, and visual inspection confirmed this finding. Lower and upper limits of the HR target training zone were determined using the Karvonen formula [5] to provide conservative estimates of these limits. The lower limit of the cardiovascular training zone for each individual is noted in Table 1. We determined the total accumulated time that the individual's HR was within the target training zone, based on the HR_10_.

Previous work used a 0–4 point scale to categorize activity levels among individuals with stroke throughout the day (activity category, AC) [10]. The same rating scale was used in the current study. Based on the activity descriptions recorded throughout the day, each period of different activity was assigned an activity level. For example, if the individual was sitting and resting (AC_0_) for a period of 3 minutes, after which he walked on a treadmill for 11 minutes (AC_4_), it was recorded that the individual performed an AC_0 _activity for 3 minutes and an AC_4 _activity for 11 minutes. For each of these two periods, e.g. 3 minutes and 11 minutes, average HR and VR values were calculated. To reflect continuous performance of an activity within a given AC, average HR and VR values were determined only if the activity was performed for 2 minutes or longer. Non-parametric methods were used to assess changes in HR and VR by AC. Kruskal-Wallis tests were used to assess changes in HR and VR with AC; individual Wilcoxon tests were used to explore significant differences between levels of AC.

## Results

### Feasibility of ambulatory monitoring

All four participants reported that the LifeShirt vest was comfortable to wear under normal clothing throughout the day. Only one individual was able to put on the LifeShirt independently (participant S1; FIM (dressing upper body) score at discharge was 7; Table 1); the other three participants required assistance. Note that electrodes for ECG monitoring were positioned and adhered by the experimenter. None of the patients reported that wearing the device restricted or otherwise impaired their movements. There were no occurrences of system or sensor problems once the system was fitted to the subject (i.e. data were collected without disruption for the 8 hour period).

### Heart rate, ventilation rate, and activity profiles: sample tracings

HR profile data gathered throughout the day indicated that the overall cardiorespiratory load was low for three of the four participants (S1, S2, S3) throughout most of the day. Including the periods of structured therapy, individuals' HRs were on average 16 bpm above their resting levels (range of 12 to 19 bpm above resting). The average HR for participant S4, including periods of structured therapy was 29 bpm above resting. However, this individuals peak demonstrated VO_2 _(8.9 mlO_2_/kg/min) was 30% lower than the average VO_2 _of the other three individuals, which suggests that this individual functioned at a higher percentage of his cardiovascular capacity when performing activities of daily living. There were important activity-related differences within each participant. To highlight these differences, a sample profile of HR and VR for S1 is presented in Figure 2, with the synchronized record of the individual's functional and physical activities. This individual's data was chosen because he demonstrated the most robust heart rate response to his physical therapy session, which may be a function of his higher FIM score results (overall score, and locomotion and upper body subscale scores).

#### Case 1 (S1)

**Figure 3:**
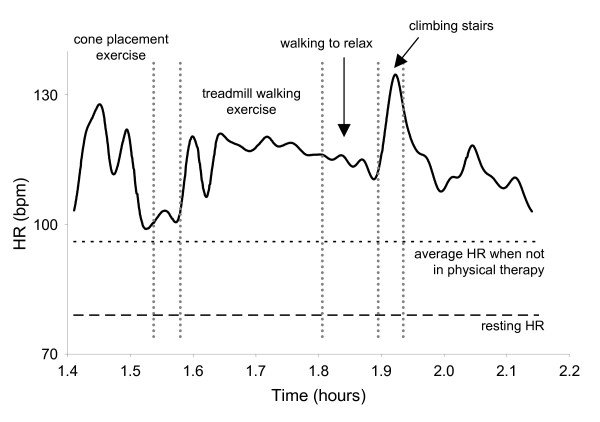
S1; profile of HR during physical therapy. The patient demonstrated clear HR responses to various activities, particularly when climbing stairs. The patient's resting HR and average HR throughout the rest of the day are indicated.

This individual demonstrated a clear heart rate response to sessions of physical therapy, but very little change in his heart rate throughout the remainder of the day. With exception of the period during which this individual was engaged in his structured physical therapy session, his average heart rate throughout the day was 95 bpm, 16 bpm above his resting HR. During physical therapy, his mean HR increased by 17%, to 111 bpm (Figure 3), and when considering only the period of time during which the individual engaged in treadmill walking and stair climbing his mean heart rate increased by 24%, to 118 bpm. He also demonstrated increases in VR during physical therapy, particularly during the cone placement and stair climbing exercises, which appeared to coincide with increases in HR. However, with the exception of the period during physical therapy, S1's heart rate varied little, despite being engaged in activities such as walking and occupational therapy.

**Figure 4:**
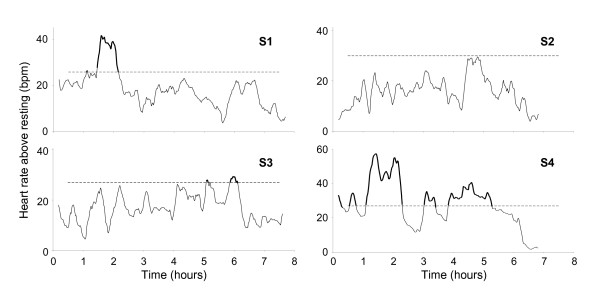
10 minute moving-average HR (HR_10_) plots for each participant; 3 of the 4 participants exceeded the minimum HR threshold to experience a cardiovascular training response associated with activities engaged in at various times throughout the day. Participants S1, S3, and S4 demonstrated HR_10 _responses that exceeded the minimum threshold for their respective training zones for totals of: 63, 38, and 253 minutes, respectively. At no point during the day did the HR_10 _of S2 reach this minimum threshold.

HR_10 _profiles are provided for each participant in Figure 4, and a description of each individual's activities along with associated HR responses (or lack of HR response) follows immediately, below.

#### Case 2 (S2)

This individual demonstrated an average heart rate throughout the day of 86 bpm (with the exception of two periods; during physical therapy and during a self-directed walking program; see below), an increase of 21% relative to his resting HR of 71 bpm. However, his HR during his physical therapy session was 88 bpm, an increase of only 2 bpm compared with his mean HR for the rest of the day, suggesting that S2 demonstrated no clear HR response to the physical therapy session. The only time during the day that this individual's HR increased notably was during a 50 minute period in the afternoon during which the individual was engaged in a self-directed walking and stretching program which was not prescribed by the physical therapist. His mean HR during this 50 minute period of self-directed activity was 98 bpm, an increase of 12 bpm, or 14%, compared with his average HR throughout the rest of the day (including the period during physical therapy). It should be noted that the patient was not being monitoring by a therapist during this period, which occurred three hours after the end of his formal physical therapy session.

#### Case 3 (S3)

Similar to S2, and in contrast to S1, S3 demonstrated no clear HR response to his structured physical therapy session. This patient's average HR was 64 bpm during physical therapy, and 65 bpm throughout the remainder of the day. S3 demonstrated small increases in HR later in the testing session (at approximately hours 5 and 6 of testing), and these elevations in HR were of sufficient duration to possibly effect a cardiovascular training response (see Heart Rate: 10 minute moving average (HR_10_), below, and Figure 4). However, these increases in heart rate did not occur during any therapy session, but, rather, while walking to the speech therapy session, and when dressing later in the day. Interestingly, even when S3 reported resting during a 50 minute period in the morning following his physical therapy session, his average heart rate was 64 bpm, which is consistent with his average heart rate throughout the day. The individual was not directly observed during that time so it is not clear if the individual was truly 'resting' or was engaged in some nominal activity which may have elevated his HR (note: the participant reported that he was intending to lie down on his bed and rest; as such, privacy was appropriately provided by the research assistant, explaining why the participant was not directly observed during this period). These findings suggest that ambulatory monitoring of physiological parameters such as HR (as well as monitoring of kinematics) may lead to more reliable reporting not only of activity but also of the intensity of activity, when compared with self-reporting.

#### Case 4 (S4)

Similar to S1, S4 demonstrated a clear HR response during physical therapy. During this period, his mean HR was 111 bpm, and increase of 80% relative to his resting HR. However, as suggested earlier, S4 demonstrated peak VO_2 _was 30% lower than that of the other three participants, which suggests that very little physical activity was required to substantially challenge this patient's cardiovascular system. This suggestion is supported by findings which indicated a marked increase in heart rate when S4 was engaged in activities such as: standing, brief periods of walking, and extended periods of sitting while eating (increase in HR of 52% compared with resting HR); and engaged in occupational therapy (increase in HR of 47% compared with resting HR).

### Heart rate: 10 minute moving average (HR_10_)

Figure 4 shows the HR_10 _profile for each individual. The heavily shaded sections of each response profile represents the period during which the individual's HR_10 _reached the cardiovascular training zone. The HR_10 _measures for 3 of the 4 participants suggest that these three individuals exceeded the minimum threshold and may have, according to the American Heart Association Scientific Statement, experienced a cardiovascular training response associated with the activities they engaged in at various times throughout the day. Participants S1, S3, and S4 demonstrated HR_10 _responses that exceeded the minimum threshold for their training zones for totals of: 63, 38, and 253 minutes, respectively. At no point during the day did the HR_10 _of subject S2 reach this minimum threshold. Of each patient's total time in the cardiovascular training zone, 83%, 0%, and 32% of this time was associated with a structured physical therapy session for participants S1, S3, and S4, respectively. The large amount of time spent by S4 in the cardiovascular training zone associated with activities such as sitting and eating, may be explained by this individual's very low cardiovascular fitness. The relatively small amount of time spent by S3 in the cardiovascular training zone might be related to his lower levels of disability as indicated by his NIHSS and FIM measures (Table 1), in addition to his relatively higher peak VO_2_. In addition, it appears that S3 was not sufficiently challenged during his physical therapy session, relative to his own cardiovascular fitness level.

### Heart rate (HR) and ventilation rate (VR): relationship to activity level

**Figure 5:**
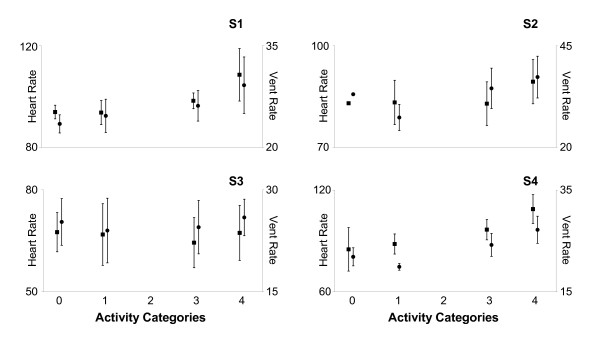
Mean (± 1 standard deviation) HR (left axis) and VR (right axis) for each participant. Statistical analysis was conducted using the data of the group as a whole. Though individual differences were observed, overall, the Kruskal-Wallis non-parametric analysis of variance revealed that both HR (black square; p = 0.0207) and VR (black circle; p < 0.0001) generally increased as AC increased HR and VR increased. For participant S2, the standard deviations for both HR and VR in AC_0 _are small and therefore the SD bars do not extend beyond the size of the symbol used in the figure. For all participants, there were no differences for both HR (p = 0.1858) and VR (p = 0.5225) between the two lowest activity levels (AC_0_, AC_1_). For HR there was no difference between AC_1 _and AC_3 _(p = 0.8874). HR for AC_4 _was significantly greater than for AC_0 _(p = 0.0105) and AC_3 _(p = 0.0396); there was no statistical difference (p = 0.094) between AC_1 _and AC_4_. VR was significantly greater for AC_3 _than for AC_0 _(p = 0.0018) and AC_1 _(p = 0.0186), and VR for AC_4 _was significantly greater than for AC_3 _(p = 0.0107).

HR and VR were compared with activity level (AC) to explore the potential relationship between the observational measure of activity level and physiological challenge, or load. Though individual differences were observed, overall, the Kruskal-Wallis (non-parametric, one-way ANOVA) test revealed that both HR (p = 0.0207) and VR (p < 0.0001) generally increased as AC increased (Figure 5). Post-hoc analysis revealed that there were no differences for both HR (p = 0.1858) and VR (p = 0.5225) between the two lowest activity levels (AC_0_, AC_1_). Also, for HR there was no difference between AC_1 _and AC_3 _(p = 0.8874). HR for AC_4 _was significantly greater than for AC_0 _(p = 0.0105) and AC_3 _(p = 0.0396), but the difference between AC_1 _and AC_4 _did not reach statistical significance (p = 0.094). VR was significantly greater for AC_3 _than for AC_0 _(p = 0.0018) and AC_1 _(p = 0.0186), and VR for AC_4 _was significantly greater than for AC_3 _(p = 0.0107). In the scale used by Bernhardt et al [10], activities in AC_2 _included 'sit supported out of bed' and 'transfer (with hoist)'. All of the participants in the current study were able to sit independently and did not require assistance with transfers. As a result, AC_2 _contained no samples (Figure 5, Table 1).

## Discussion

The purpose of this study was to: 1) examine the physical activity levels and associated cardiorespiratory responses of individuals with stroke during normal daily activities which included their structured physical therapy sessions, and 2) examine the relationship between a previously reported activity level classification with measured physiological responses to daily activity (heart rate, ventilation rate). We used a commercially available wearable ambulatory physiological monitoring system. This study linked measured physiologic change with specific daily activities including activities associated with structured rehabilitation sessions, as well as the activities and times when the individuals were not in therapy.

Importantly, the findings of this study provide direct physiologic evidence to support the suggestion that individuals with stroke are generally inactive throughout the day, which is consistent with observational reports in the literature [10,11]. Little information regarding the activity patterns of individuals with stroke throughout the day is available. Though Bernhardt et al. [10] demonstrated that individuals in the acute phase of recovery following stroke are generally inactive according to a subjective scale rating the therapeutic level of various activities from 0 (inactive) to 4 (highly therapeutic), the findings of the current study suggest that, among individuals in the subacute stage of recovery, even activities included in the categories of highest therapeutic relevance (e.g. walking) may not load the cardiorespiratory system sufficiently to elicit a training effect. Although both HR and VR generally increased with the subjectively rated AC, large individual differences in these relationships, as well as large ranges in the measures of HR and VR within each AC, for each individual. These findings suggest that physiological load cannot be assessed directly from AC. For example, S1 and S4 demonstrated marked differences in mean HR and VR across the AC levels (Figure 5). S2 demonstrated similar differences across AC levels 1, 3, and 4, though both HR and VR were greater for AC_0 _than for AC1. S2 spent only 2% of the testing session in the AC_0 _category (see Table 1), and this period may have been marked by an elevated HR and VR for reasons other than physical activity (i.e. anxiety or other stress). S3 demonstrated no apparent change in HR or VR across the AC categories. In addition to individual differences in the physiological response to activity across the individuals, each patient demonstrated large ranges in the measures of HR and VR within each AC, particularly for AC_3 _and AC_4_. For S1, HR ranged between 91 and 102 bpm during AC_3 _activities, and between 90 and 130 during AC_4 _activities. For S2, HR ranged between 74 and 95 bpm during AC_3 _activities, and between 81 and 101 bpm during AC_4 _activities. The other two participants demonstrated similar HR responses. The average HR range during AC_3 _activities across the four individuals was 18 bpm; during AC_4 _activities, the average HR range was 32 bpm. Furthermore, S1 demonstrated HR responses adequate to elicit a physiological training effect (i.e. HR greater than the minimum threshold for a training effect according to the American Heart Association scientific statement) for less than 50% of the time this individual spent in AC_4 _'highly therapeutic' activities. During AC_3 _'moderately therapeutic' activities, this same individual's HR did not enter the training zone at all. Clearly, an observational measure of activity level does not adequately describe the physiological load, or potential benefit, of individual activities, and addition of physiological parameters such as HR or VR are needed to assess the physiological load of activity for individuals. Ambulatory monitoring of physiological load during activity provides the capacity to assess the aerobic challenge associated with activity and adjust the intensity of activity on a person-to-person basis.

While previous work has inferred the therapeutic relevance of physical activity based on the expert opinion of experienced clinicians, the current study has added direct physiological measurement of the physiological load the activity to the understanding of the (potential) health benefits associated with the activity. This additional information available through the use of the physiologic monitoring has provided three important insights. First, and consistent with work by MacKay-Lyons and Makrides [5], the physiological load experienced by individuals during structured therapy sessions may not be sufficient to elicit a cardiovascular benefit or training effect. Second, tremendous individual differences exist in the individual's physiological response to physical activity during therapy and throughout the day. Third, even during activities which are deemed by expert opinion to be highly therapeutic, large ranges in measures of physiological response (i.e. heart rate, ventilation rate) suggest that these activities do not necessarily provide a cardiovascular training effect. These insights confirm that it is imperative that ambulatory physiological measurement systems (i.e. wearable heart rate monitors) be used during physical therapy sessions not only to ensure the safety of the patient, but also (and likely more commonly) to ensure that the patient is engaged with sufficient intensity to challenge the cardiovascular system to the point of training effect. Furthermore, the findings of the current study underscore the need to better understand the nature of the physical activities engaged in by individuals throughout the day, such as the type of activity, the duration that specific activities are performed, and the intensity of the activity in terms of the cardiorespiratory response. Ambulatory physiological monitoring of individuals with stroke throughout the day may provide a method of influencing individual activity profiles on a day-to-day basis and eventually via a method of real-time monitoring and prompting.

Activities engaged in by the individuals throughout the day were categorized according to a previously established method using observation techniques to infer therapeutic value of physiologic loads associated with activity [10]. The results suggested that the four participants in the current study were engaged in activity that was deemed non-therapeutic for, on average, slightly more than 50% (range of 23 to 65%) of the day, which is consistent with the report of Bernhardt and colleagues [10]. The individuals in the previous study spent 28% of the day engaged in minimally therapeutic activities (i.e. sitting supported out of bed). The individuals in the current study did not perform any activities that were considered to be in the minimally therapeutic category. Therefore, it seems that the individuals in the current study had a greater volume and extent of activity in categories of higher therapeutic relevance due, in part, to their higher functional capacity. For example, they were all capable of sitting unsupported, and therefore spent a larger percentage of the day, according to this scale, engaged in moderately and highly therapeutic activity (50% of the day, versus 12.8% in the previous study). The previous work by Bernhardt [10] examined individuals with stroke at an early stage of recovery while the current study explored activity profiles of in-patients who were later in their stage of recovery (four to eight weeks after stroke). It is unlikely that individuals able to ambulate independently (with aids), such as those who participated in the current study, would find sitting unsupported substantially challenging from a sensorimotor perspective or in terms of cardiovascular load, and therefore the recovery time differences may explain the increase in activities which, according to this scale, would be considered therapeutically-relevant if using the activity scale. These findings suggest that development of an alternate activity level scale designed specifically for individuals at later stages of recovery following stroke might be useful and more discriminative in assessing the physiological challenge of various daily activities.

A limitation of this study was sample size; a research assistant was required to spend 8 to 9 hours observing each participant, limiting the feasible number of participants, and limiting data collections to a single day. Therefore, the sample of participants included individuals who varied greatly in age and neurologic impairment, in order to explore in a case-study approach the level of activity among patients with stroke, and the relationship between activity level classification and continuously sampled physiological response. The development of movement assessment capability (e.g. accelerometers) and validation of the discriminative capacity of such measurements (to distinguish movement profiles) is essential to improve the practical application of this approach to remove time and cost constraints imposed by the necessity of a research assistant to manually document participant activities all day long. Such remote measurement of movement, as opposed to relying on observation, would also help to counter limitations associated with privacy and observation. In addition, it is possible that the participants may have altered their normal daily activities, or altered the level of effort provided during various tasks as a result of being observed throughout the day. It should be noted, however, that one might have anticipated an increase in relative activity under such a scenario and in the case of the present individuals they were characterized by relatively low levels of daily activity.

This study confirms and extends the results of previous research providing a detailed view of the activity patterns of individual patients with stroke and the associated physiological response throughout the day. First, the activity level of individuals with stroke during structured therapy sessions may not be of sufficient physiological challenge to elicit a cardiovascular training effect. Second, they appear to be relatively inactive throughout the day, and simple observation of their physical activity may not assess the therapeutic relevance of the activity. Third, we have associated a measure of physiological challenge with the individual's activities of daily living. Future research will examine methods of influencing the activity level of individuals with stroke in the rehabilitation hospital and community. Incumbent in that research will be the development of technology which will associate kinematic measurements with physiologic data. Such developments will facilitate inclusion of a larger sample size by autonomously providing a context of activity to the physiologic measure, reducing the cost of data gathering and enhancing feasibility. Data acquisition systems built on emerging sensor technologies will provide the understanding of the individual's activity necessary for meaningful interpretation of the physiologic response to activity throughout the day (and night). Information related to activity obtained at times outside of structured therapy sessions may serve to provide important insight regarding the individual's status not otherwise available to the therapist. In addition, these developments will allow precise measurement of function and intensity of activity in the community, promoting evidence-based therapeutic practice following discharge from the daily therapy program or rehabilitation hospital.

## Competing interests

None of the authors have a conflict of interest related to the publication of this manuscript. While Vivometrics donated the use of the LifeShirt system, Vivometrics had no input to the design of the research, the collection of data, the analysis of data, or the development of this manuscript.

## Authors' contributions

WHG, KFZ, DB, and WEM conceived of the study and participated in its design and coordination and helped to draft the manuscript. WHG, KMS, and AT recruited study participants and collected the data. All authors read and approved the final manuscript.
